# Population genetics of the main population of brown bears in southwest Asia

**DOI:** 10.7717/peerj.5660

**Published:** 2018-09-21

**Authors:** Hüseyin Ambarlı, Deniz Mengüllüoğlu, Jörns Fickel, Daniel W. Förster

**Affiliations:** 1 Department of Wildlife Ecology and Management, Düzce Unviersity, Düzce, Turkey; 2 Leibniz Institute for Zoo and Wildlife Research, Berlin, Germany; 3 Institute for Biochemistry and Biology, University of Potsdam, Potsdam, Germany

**Keywords:** *Ursus arctos*, Microsatellite, Conservation, Anatolia, Isolation, Source population, Noninvasive sampling, Rubbing tree, Turkey

## Abstract

Genetic studies of the Eurasian brown bear (*Ursus arctos*) have so far focused on populations from Europe and North America, although the largest distribution area of brown bears is in Asia. In this study, we reveal population genetic parameters for the brown bear population inhabiting the Grand Kaçkar Mountains (GKM) in the north east of Turkey, western Lesser Caucasus. Using both hair (*N* = 147) and tissue samples (*N* = 7) collected between 2008 and 2014, we found substantial levels of genetic variation (10 microsatellite loci). Bear samples (hair) taken from rubbing trees worked better for genotyping than those from power poles, regardless of the year collected. Genotyping also revealed that bears moved between habitat patches, despite ongoing massive habitat alterations and the creation of large water reservoirs. This population has the potential to serve as a genetic reserve for future reintroductions in the Middle East. Due to the importance of the GKM population for on-going and future conservation actions, the impacts of habitat alterations in the region ought to be minimized; e.g., by establishing green bridges or corridors over reservoirs and major roads to maintain habitat connectivity and gene flow among populations in the Lesser Caucasus.

## Introduction

The brown bear (*Ursus arctos* Linnaeus, 1758) is a widely distributed Holarctic old world species. Although the main distribution of the species is in Asia, genetic studies have largely focused on Europe and North America (Swenson, Taberlet & Bellemain, 2011). Currently, there are only a few genetic studies from the Asian portion of the species’ distribution (Bellemain et al., 2007; Murtskhvaladze, Gavashelishvili & Tarkhnishvili, 2010; Çilingir et al., 2016). The main brown bear population in southwest Asia is in northeastern Anatolia (hereafter using the term Anatolia for the Asiatic part of Turkey), consisting of more than 2,000 individuals (Ambarlı, Ertürk & Soyumert, 2016). This population is characterized by a higher abundance (Ambarlı, 2016) and a higher mtDNA diversity (Çilingir et al., 2016) than the neighboring, and mostly isolated, populations in the Lesser Caucasus parts of Georgia, Armenia, and Azerbaijan (Lortkipanidze, 2010). Additional brown bear populations from Lebanon, Syria and Iraq have recently become extinct (IUCN, 2016). The northeastern Anatolian population can thus be considered the main source population in southwest Asia (Ambarlı, 2016). In order to protect this population and properly manage the species in this region, it is essential to gain greater insight into its ecology and population genetics, particularly when the continued habitat fragmentation and destruction of the natural ecosystems of Turkey are considered (Şekercioğlu et al., 2011).

**Figure 1:**
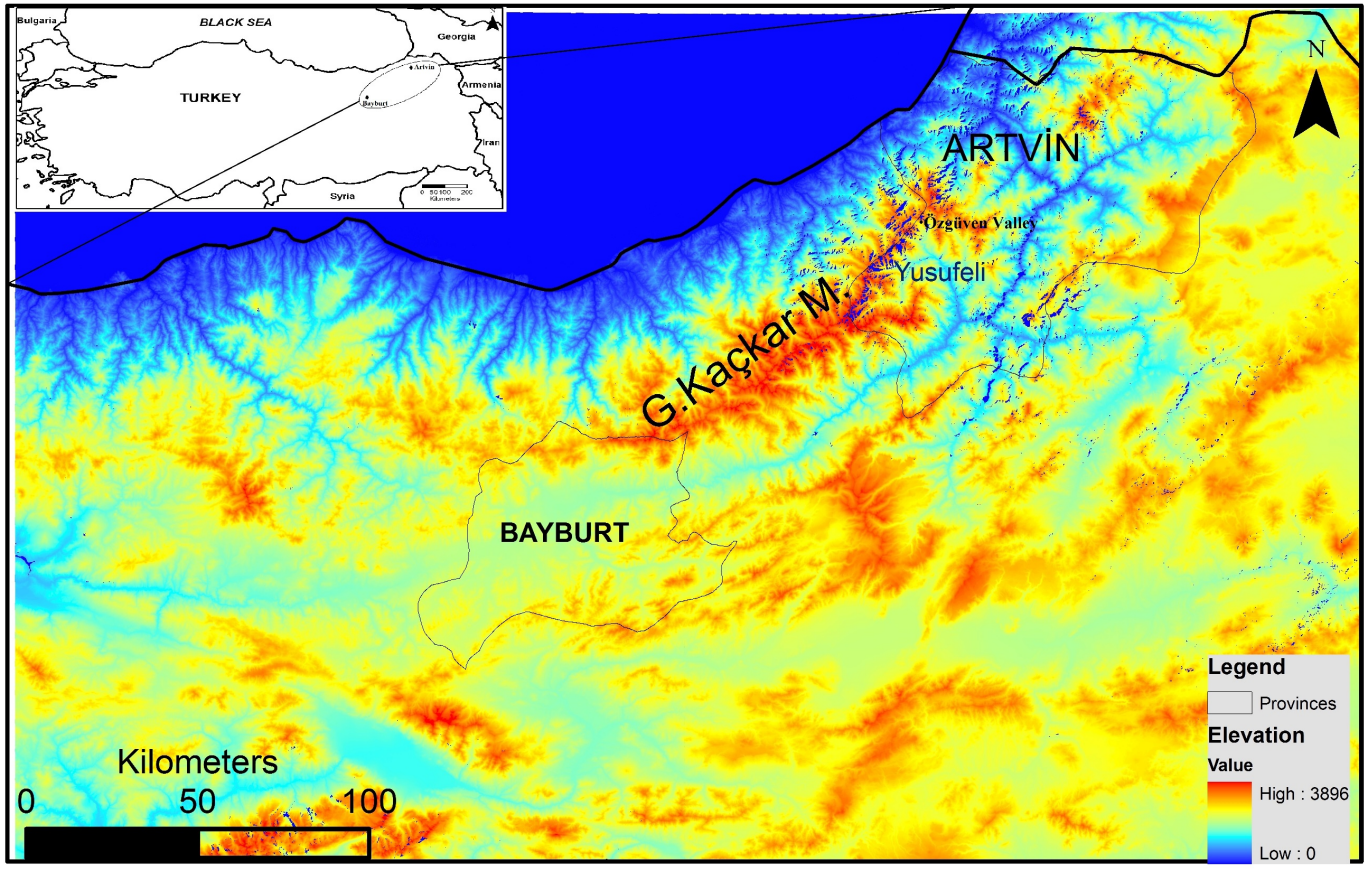
Map of the study area.

In spite of the recent increase in anthropogenic pressure threatening the survival of brown bears in Turkey, the northeastern Anatolian population still appears viable (Ambarlı, Ertürk & Soyumert, 2016). However, thorough assessment regarding its future viability is not yet possible because detailed population parameters (ecological, genetic and demographic) are still unknown. The Grand Kaçkar Mountains (GKM) are located in northeastern Anatolia, and are home to the largest intact brown bear population in southwest Asia (Fig. 1). The GKM form a natural barrier between inner Anatolia and the Black Sea Region, and provide continuous pristine habitat (e.g., natural old forests) for large carnivores in the region.

However, there are increasing threats from growing touristic activities and numerous planned hydro-electrical power plants (HEPP). For example, 28 big dams on Çoruh River (eastern part of GKM) and more than 1,000 small HEPP are to be constructed in the montane area of the Black Sea and Eastern Anatolia Regions at elevations between 750–2,500 m above sea level (Atak & Öztok, 2013). The construction of HEPPs in this region (325 HEPPs are already constructed, and 400 are under construction, and 350 more HEPPs are planned; http://www.enerjiatlasi.com/hidroelektrik/) requires substantial logging to build new roads and high-voltage transmission lines at these high altitudes (Şekercioğlu et al., 2011). This logging destroys areas of pristine habitat currently used by bears for hibernation and cub rearing (Ambarlı, Ertürk & Soyumert, 2016).

Furthermore, the flooding of large areas by new water reservoirs and the construction of new big dams in the eastern GKM will likely hamper the movement of bears between GKM and the Lesser Caucasus Mountains in Georgia (Fig. 1). A habitat modeling study examining the consequences of these reservoirs and dams for the local fauna in the GKM reported that habitats of large mammal species will become fragmented, causing the isolation of populations (Özdemirel, Turak & Bilgin, 2016). An additional concern is that the high-voltage transmission lines and pylons needed for HEPPs will further contribute to habitat fragmentation in the region (Balkenhol & Waits, 2009). Wildlife refuges at higher altitudes are further threatened by extended road construction above 2,000 m to boost “ecotourism activities” (WWF-Türkiye, 2015; Ambarlı, Ertürk & Soyumert, 2016). These roads starting in the middle of the Black Sea Region and stretching for more than 2,500 km to the Georgian border, will further fragment pristine habitats and enhance their erosion.

An additional concern is human-induced fatalities, because bears are frequently involved in human-wildlife conflicts in the region and are thus the target of retaliatory poaching and trophy hunting (Ambarlı, Ertürk & Soyumert, 2016). After a hiatus of more than 10 years, the Board of National Park Directors of Turkey has recently reissued a bear trophy hunting quota, and decreased the fine for illegal poaching from $3,500 to $800, despite the lack of reliable sustainability information (e.g., by using demographic and genetic tools).

Habitat fragmentation and population isolation has been shown to result in low within-population genetic diversity in American grizzly bears (Paetkau et al., 1998). If large mammals in northeastern Anatolia become subjected to increased habitat fragmentation and population isolation, as predicted by modeling studies (Balkenhol & Waits, 2009; Özdemirel, Turak & Bilgin, 2016), then it is likely that the brown bear population in the GKM will also suffer a loss of genetic diversity (Paetkau et al., 1998; Frankham, 2005). This would be further exacerbated by human-induced fatalities resulting from increased hunting quotas, as well as by the reduced deterrent for poaching (significantly reduced fines). Considering the multiple threats to brown bears and their habitat outlined above, the fate of the main population in northeastern Anatolia over the next decades is uncertain.

In this study, we focus on the bear population of the Grand Kaçkar Mountains (GKM; Fig. 1) in northeastern Anatolia. In this mountain chain, connected to the Lesser Caucasus, several bear subpopulations are distributed in habitats separated by high mountains or fast-flowing rivers in deep and rugged valleys. It is currently not known if there is gene-flow between these subpopulations, and if so, to what degree. Thus, the aims of this study were (i) to characterize the current level of genetic variability of the GKM population, and (ii) to determine if geographic features of the Grand Kaçkar Mountains impact dispersal sufficiently to impede gene flow between subpopulations in the area. The study presented here is the first one using nuclear loci to assess levels of genetic diversity of the main brown bear population in Turkey. By doing so, we will also generate a “genetic baseline” needed to measure future impacts of anthropogenic habitat alterations and poaching in this region.

## Materials & Methods

### Samples

We collected 154 samples from the Grand Kaçkar Mountains (GKM) region in two neighboring Turkish provinces, Artvin and Bayburt (Fig. 1; Table 1), respectively located on the eastern and the southwestern edges of the GKM. Both are small montane provinces in the Lesser Caucasus Region characterized by steep mountains and large fast-flowing rivers. We chose to sample in these provinces because current dam construction activities (including new roads and forest logging) may reduce connectivity between eastern and western GKM (sub)populations even further in the near future. Obtaining samples from these locations enabled us to assess population connectivity between areas separated by up to 300 km, a distance near the limit of the homing ability of brown bears (Conover, 2001). The bear population in Artvin is connected to bear populations in Georgia, and the bear population in Bayburt is connected to bear populations in Eastern and Central Anatolia (Ambarlı, Ertürk & Soyumert, 2016).

**Table 1:** Sample details.

Province	Period	Size of area sampled (km^2^)	No. of tissue samples	No. of hair samples	No. of hair samples successfully genotyped^a^	No. of samples used for individual identification^b^	No. of unique genotypes identified^c^	No. of samples used in genetic analyses^c^^,^^d^
Artvin	2008–2014	2,425	7	127	56	63	42 + 1	36 + 1
								
Bayburt	2012	2,280	–	20	8	8	5 + 1	5 + 1
								
	Total	4,705	7	147	64	71	48	42

**Notes.**

a Here ‘successfully genotyped’ indicates that at least eight microsatellite loci amplified successfully.

b Unique genotypes were identified using data from both tissue and hair samples (see Methods for details).

c Here ‘ +1’ is used to indicate that one unique genotype was observed in both provinces.

d Genetic analyses were conducted using genotypes with data missing for only one of ten microsatellite loci; again, ‘ +1’ is used to indicate that one unique genotype was observed in both provinces.

Noninvasive genetic sampling was carried out from 2008 to 2014 (from May to October). In the Artvin province, we collected hair samples from rubbing trees and power poles impregnated with creosotes (*N* = 105), corral hair-traps (*N* = 7), and from barbed wires placed on rubbing trees (*N* = 12), at altitudes between 700–2,500 m above sea level (Table 1). Additionally, we used three invasive hair samples from captured bears (*N* = 3). Samples from the Bayburt province were collected from rubbing trees and power poles (*N* = 20). Hair samples were placed in paper envelopes without contacting human skin and were then stored at room temperature in zip lock bags with silica gel (Roon, Waits & Kendall, 2003). In addition, fresh tissue samples (*N* = 7) were obtained from bears captured in the Artvin province between 2010 and 2011 (Permission No:B.23.0.DMP.0.13.02–445.05-36125, Ministry of Forestry and Water Affairs) (Ambarlı, 2012), and were stored in 98% ethanol**.** In total, we collected 147 hair samples (Noninvasive *N* = 144, invasive *N* = 3) (Artvin *N* = 127, Bayburt *N* = 20) and seven tissue samples (Artvin *N* = 7) (Table 1).

### DNA extraction and genotyping

For both hair and tissue samples, DNA was extracted using a commercially available kit (GEN-IAL GmbH, Troisdorf, Germany) following the manufacturer’s instructions without any modifications.

We genotyped all samples at 10 microsatellite loci: G10C, G10X, UarD1585, UarT739, UarD3139, UarD3684, G1D, Mu05, Mu23, Mu50, and at the sexing locus SRY (primer details in Table S1). One primer of each primer pair was 5′  labeled with a fluorescent dye (6-FAM, HEX, or NED), so that differently labeled primer pairs could be used in a multiplex approach for genotyping. Loci were amplified using the Qiagen Multiplex PCR Kit (Qiagen, Hilden, Germany) in three multiplexes of 10 µL final reaction volume, following the manufacturer’s recommended conditions (multiplexes detailed in Table S1).

Following Miller, Joyce & Waits (2002), we used a Maximum Likelihood approach for genotyping noninvasively collected samples: each sample was genotyped in parallel (*N* = 2 replicates per sample) and if a mismatch occurred between replicates, results were discarded and genotyping was repeated on a new DNA extraction of that sample, resulting in 2–4 replicates per sample. The low number of hairs collected per sample limited us to two DNA extractions per sample. Thus, if a mismatch occurred in the second round of parallel genotyping, the sample was discarded.

As bears are highly mobile, we had to consider multiple sampling from a single individual. To remove such bias, we looked for matching genotypes using the R package (R Core Team, 2015) *allelematch* (v2.5; Galpern et al., 2012) applying the option ‘alleleMismatch=2’. Matching genotypes were always assigned to the same individual, mismatching genotypes were only assigned to the same individual if either one of the following criteria was met: (i) only one allele difference was observed between genotypes and could be attributed to large allele drop-out or allele-size shift due to stuttering (*N* = 3), or (ii) two allele differences between genotypes were observed but could be attributed to large allele drop-out, allele-size shift or missing data (*N* = 6). Genotypes from tissue samples were included in this analysis.

### Genotypic analyses

The probability of null alleles being present in the data set was assessed using Micro-Checker (v.2.2; Van Oosterhout et al., 2004). To assess if the number of loci and alleles was sufficient to discriminate individuals, we estimated the cumulative values of the unbiased probability of identity (*P*_*IDunb*_) and of the probability of identity given siblings (*P*_*IDsib*_) using the software package Gimlet (v.1.3.3; Valière, 2002).

We examined the genotypes of the GKM bear population using the following software packages: Popgene (v.1.32; Yeh et al., 1997) to test for deviation from Hardy–Weinberg equilibrium, and to estimate observed heterozygosity (*H*_*O*_) and expected heterozygosity (*H*_*E*_), and fstat (v.2.9.3.2; Goudet, 2002) to estimate the inbreeding coefficient *F*_*IS*_.

Genetic structure among sampling sites was examined using multiple approaches because these can differ in their assumptions about markers or populations, such as random mating, absence of selection or absence of mutation. We thus included individual-based analyses that do not make such assumptions, as well as analyses that infer population genetic structure using allele frequencies that do make such assumptions.

The following analyses are individual-based and are not be impacted by, for example, uneven sampling of populations or deviation from HWE. The R package *memgene* (v.1.0; Galpern et al., 2014) was used to calculate the proportion of shared alleles among samples (following Bowcock et al., 1994), and the package *ape* (v.5.1; Paradis, Claude & Strimmer, 2004) to construct unrooted neighbor-joining trees from the distance matrix. We used *adegenet* (v.2.0.1; Jombart, 2008) to conduct principal component analyses (PCA). *PopGenReport* (v.2.1; Adamack & Gruber, 2014) was used to calculate pairwise genetic dissimilarity between samples (following Kosman & Leonard, 2005) and to plot these values against the geographic distance between samples. This allowed us to retain information regarding the resampling of individuals (i.e., unique genotypes) in a geographic context, as it was conducted on the dataset consisting of 71 samples that had been scored at a sufficient number of microsatellite loci (Table 1). Thus, for example, the genotypes that had been detected at more than one location could be included, together with spatial data regarding sampling location. Native functions of R were also used to plot graphs.

The following analyses infer population genetic structure using allele frequencies that do make such assumptions, and can be impacted by deviations from these assumptions (e.g., if they are not in HWE, by Wahlund effect). Arlequin (v.3.5.2.1; Excoffier & Lischer, 2010) was used to calculate population pairwise *F*_ST_; significance was tested by permuting individuals between populations (10,000 permutations).

We used Bayesian inference implemented in the software Structure (v.2.3.4; Pritchard, Stephens & Donnelly, 2000; Falush, Stephens & Pritchard, 2003) to estimate the number of genotypic clusters in our study area. We used the admixture model as prior and ran the software for 600,000 steps following a burn-in of 200,000 steps. The most likely number of genotypic clusters (*K*) was evaluated in a range from *K* = 1 to *K* = 6 and was assessed in ten independent runs per *K.* We also used Bayesian inference implemented in the software tess (v.2.3; Chen et al., 2007) to infer population structure, and included spatial data regarding sampling location. We used the admixture model and ran the software for 100,000 sweeps, discarding the first 50,000 as burn-in. We ran tess 100 times for each *K* in a range from *K* = 2 to *K* = 6, and averaged results (per *K*) over the 20% runs with the lowest Deviance Information Criterion (DIC). Results were visualized using clumpp (v.1.12; Jakobsson & Rosenberg, 2007) and distruct (v.1.1; Rosenberg, 2004).

To examine the impact of sampling date and sample source on genotyping success, we compared results for hair samples collected from different noninvasive sources (*N* = 144) and years (2008–2014) using Chi-square and contingency tables. Understanding how age and source of samples impact genotyping success can inform improving sampling strategy for further studies.

## Results

### Microsatellite variation

The microsatellite loci used were all highly polymorphic, with a range of seven to ten alleles (Table 2). In total, 64 out of the 147 hair samples (43.5%) yielded data for at least eight out of the ten microsatellite loci (Table 1). Applying the criteria outlined above (Materials & Methods), we scored 48 unique genotypes among these 64 hair and the seven tissue samples (71 samples total). Based on the SRY data, these consisted of 40 males (83.3%) and eight females (16.7%). However, we also found that SRY worked for a tissue sample from a captured female bear and for one hair sample (H58) that had four exact matches to a female (Samples H20, H51, H110, H114, see dataset in the Supplemental Files). We detected 14 unique genotypes (12 males and two female genotypes) during a single sampling period (2010) in a small part of the Artvin sampling area (<100 km^2^).

**Table 2:** Summary of genotyping results at 10 microsatellite loci for the Anatolian GKM population.

Locus	*N*_*allele*_	*H*_*O*_	*H*_*E*_	*HWE*	*Null*_*ch*_	*F*_*IS*_
Mu50^a^	10	0.81	0.85	n.s.	0.03	0.063
UarT739^a^	7	0.71	0.73	n.s.	0.02	0.046
G10X^a^	9	0.76	0.76	n.s.	−0.01	0.009
UarD3139^a^	9	0.73	0.82	n.s.	0.06	0.119
UarD3684^a^	8	0.67	0.75	n.s.	0.06	0.121
G10C	9	0.66	0.83	^*^	0.12	0.219^*^
G1D	8	0.59	0.80	^*^	0.15	0.278^*^
UarD1585	10	0.62	0.86	^*^	0.16	0.290^*^
Mu05	8	0.50	0.69	n.s.	0.16	0.283^*^
Mu23	7	0.61	0.80	n.s.	0.14	0.252^*^
						
*over five loci without null alleles*^a^	*8.6*	*0.74*	*0.78*			*0.072*

**Notes.**

Number of alleles (*N*_*allele*_), observed (*H*_*O*_) and expected (*H*_*E*_) heterozygosity, deviation from Hardy–Weinberg equilibrium (*HWE*, n.s., not significant; *, significant at *P* < 0.05), estimated frequency of null alleles (*Null*_*ch*_) following Chakraborty et al. (1992), inbreeding coefficient (*F*_*IS*_; *, significant at *P* < 0.05).

a Loci used in comparison to other brown bear populations (Table 5).

In order to reduce the impact of missing data on downstream analyses, we discarded six unique genotypes because there were missing data at more than one locus (Table 1). Out of the remaining 42 unique genotypes, 36 were detected in the Artvin province, five were detected in the Bayburt province, and one genotype was observed in both provinces (Table 1).

Evaluation of microsatellite allele distribution data using MicroChecker revealed that five loci had a significant probability for the presence of null alleles (G10C, UarD1585, G1D, Mu05, and Mu23; Table 2). These five loci also displayed significant levels of inbreeding (measured as *F*_IS_), and three of them (G10C, UarD1585, and G1D) showed significant deviation from Hardy–Weinberg equilibrium (Table 2).

While inclusion of these loci did not alter the results of analyses on population structure, we restricted analyses to the five remaining loci (Mu50, UarT739, G10X, UarD3139, UarD3684) as these were still capable of distinguishing 41 out of the 42 unique genotypes identified using the full set of markers. Reducing the number of loci resulted in the cumulative estimates of ‘probability of identity’ dropping from *P*_*IDunb*_ = 2.7 × 10^−13^ and *P*_*IDsib*_ = 5.2 × 10^−5^ (10 loci) to *P*_*IDunb*_ = 8.7 × 10^−7^ and *P*_*IDsib*_ = 7.7 × 10^−3^ (5 loci).

Over all genotypes (*N* = 42), expected and observed heterozygosity was high (*H*_*E*_ = 0.78, *H*_*O*_ = 0.74; Table 2). When considering genotypes detected in only one province (i.e., excluding the genotype found in both provinces), we observed virtually identical values for genotypes from the Artvin province (*N* = 36, *H*_*E*_ = 0.79, *H*_*O*_ = 0.74), and slightly lower values for genotypes from the Bayburt province (*N* = 5, *H*_*E*_ = 0.68, *H*_*O*_ = 0.72).

### Population structure

**Figure 2:**
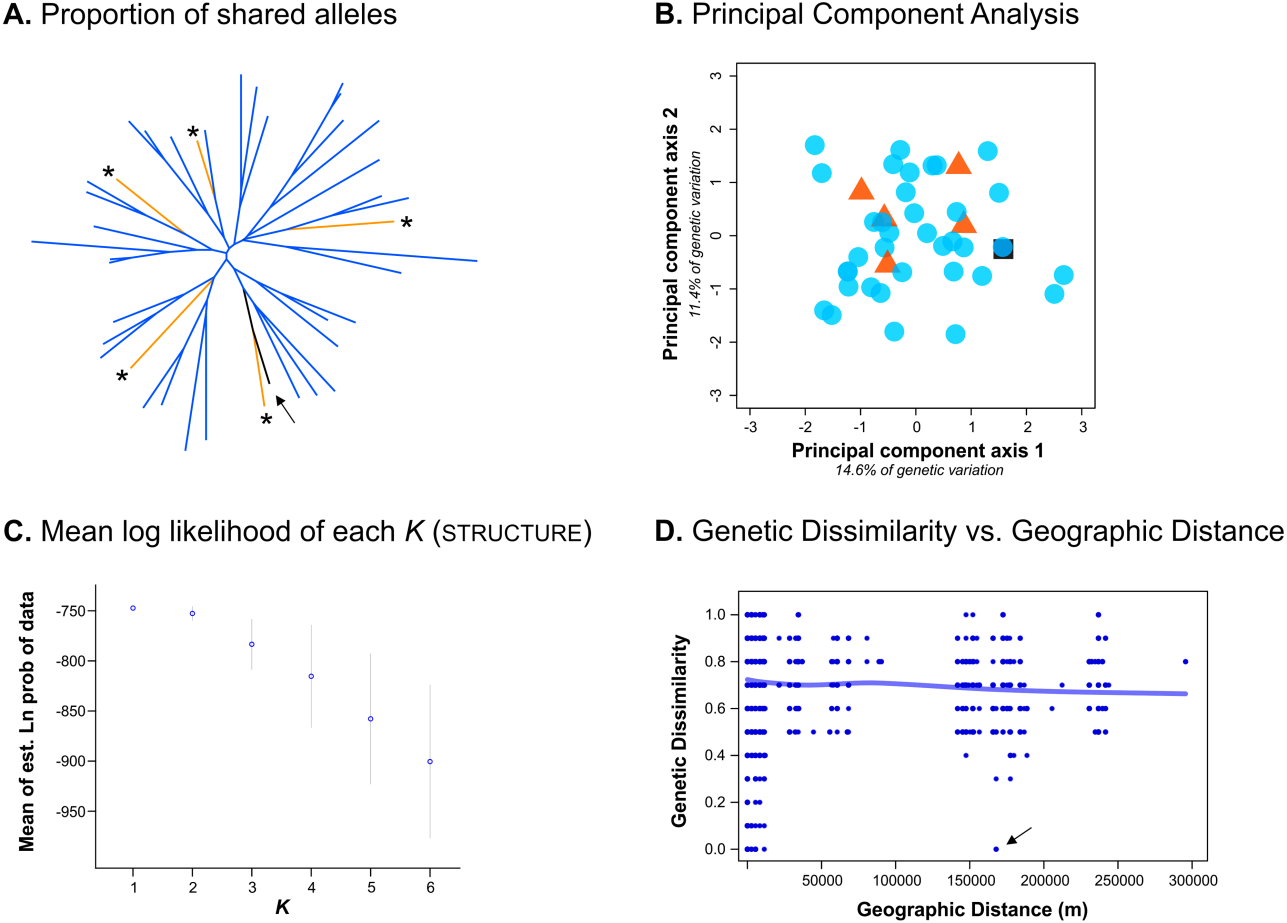
Analyses conducted to examine genetic population structure. (A) The proportion of shared alleles between individuals (following Bowcock et al., 1994), visualized as an unrooted neighbor-joining tree. Sample origin is indicated by colour; genotypes from the Bayburt province are additionally indicated by an asterisk ‘*’; the genotype found in both provinces is indicated by an arrow. (B) Principal component analysis (PCA). Sample origin is indicated by colour; genotypes from the Artvin province are represented by blue circles; genotypes from the Bayburt province are represented by orange triangles; the genotype found in both provinces is represented by a black square. (C) Structure results; plot of mean log likelihoods for *K* = 1 to *K* = 6. (D) Plot of genetic dissimilarity (following Kosman & Leonard, 2005) versus geographic distance (m). The line represents the running average of genetic dissimilarity.

Despite only obtaining a moderate number of genotypes from the Bayburt province, we nevertheless attempted to resolve brown bear population structure in our study area. However, excluding the genotype found in both provinces, we found no genetic differentiation between the brown bear populations from the Artvin and Bayburt provinces (*F*_ST_ = 0.004, *P* = 0.47; *N* = 41). When examining the proportion of shared alleles between individuals (following Bowcock et al., 1994; *N* = 42), no clustering of genotypes based on sample origin was apparent (Fig. 2A). Similarly, the principal component analysis (PCA; *N* = 42) did not show a clustering of genotypes based on sample origin (Fig. 2B). Bayesian inference using Structure (*N* = 42) suggested the presence of a single population (Fig. 2C; for Structure plots of *K* = 1 to *K* = 6 see Fig. S1).

To take spatial information into account, we firstly examined genetic dissimilarity versus geographic distance (Fig. 2D). Within the geographic range of the study, we found both samples with no genetic dissimilarity at short distances (up to ∼15 km apart, resampled genotypes within provinces), and at long distances (160 km, the genotype detected in both provinces, indicated by an arrow in Fig. 2D). Pairwise values among samples from the two different provinces (geographic distance of ∼150 km to 300 km) were mostly on the same order as pairwise values for samples from the same province (geographic distance of 0 km to ∼100 km). Thus, genetic dissimilarity did not increase with increasing geographic distance at the spatial scale of our study (horizontal line in Fig. 2D).

We also assessed population structure by Bayesian inference implemented in tess, incorporating spatial data. As this software does not calculate the likelihood of all individuals belonging to a single population (*K* = 1), we conducted the analysis for *K* = 2 to *K* = 6. Neither the Deviance Information Criterion (DIC) nor the plots of individual cluster membership provided unambiguous evidence for a population subdivision. Only up to five genotypes had a cluster membership probability above 0.8 (for *K* = 2) in any analysis (Fig. S2).

### Source of hair samples

Hair samples collected from rubbing trees (with or without barbed wire) yielded DNA extracts that performed significantly better for genotyping than did samples collected from power poles treated with creosotes (Chi-square = 15.81, *Df* = 1, *P* < 0.001) regardless of the collection year (Tables 3 and 4). Overall, we observed a significant difference in genotyping success among different noninvasive sources of hair samples (Chi-square = 19.09; *Df* = 3; *P* = 0.0026) (Table 4).

**Table 3:** Genotyping success rates for hair samples (*N* = 147) obtained in different time periods between 2008 and 2014.

	2008	2009	2010	2011	2012	2014	Total
Successfully genotyped	11	1	16	12	17	7	64
Failed	23	14	11	18	15	2	83
*Success rate %*	*32.4*	*6.7*	*59.3*	*40.0*	*53.1*	*77.8*	*43.5*

**Table 4:** Genotyping success rates for non-invasively collected hair samples (*N* = 144) from different sources in the field.

	Poles	Natural trees	Barbed wire on trees	Hair trap on the ground	Total
Positive	19	29	10	3	61
Negative	53	24	2	4	83
*Success rate %*	*26.39*	*54.72*	*83.33*	*42.86*	*42.36*

## Discussion

Measuring current levels of genetic variation is an essential aspect of conservation genetics (Paetkau et al., 1998; Frosch et al., 2014; Tsaparis et al., 2015; Bull et al., 2016), as it provides the data necessary for measures assuring a sustainable population in the future. In this study, we focused on the bear population inhabiting the Grand Kaçkar Mountains (GKM) in the north east of Turkey, which comprises the main source population in Eastern Turkey and the Lesser Caucasus (Ambarlı, 2016).

Using both tissue and hair samples collected between 2008 and 2014 (Table 1), we were able to detect at least 48 bears in the Artvin and Bayburt provinces. Previous bear population density estimates in the Artvin province suggested that this region had one of the highest bear densities in the world (Ambarlı, 2012). We can also infer a high density of bears in this region from the 14 unique genotypes detected during a single sampling period (2010) in the Özgüven valley of the GKM (Fig. 1). At least four females with cubs were also present in the area (Ambarlı, 2012), and two went un-sampled in our study. Taking these two females and undetected independent individuals into account, these numbers can correspond to a similarly high density (about 20 bears/100 km^2^) as reported previously by Ambarlı(2012).

### Genetic diversity

We found high levels of allelic variation among bears in our study area (*H*_*E*_ = 0.78, *H*_*O*_ = 0.74; Table 2), which were also apparent when the two areas were examined separately (Artvin province: *N* = 36, *H*_*E*_ = 0.79, *H*_*O*_ = 0.74; Bayburt province: *N* = 5, *H*_*E*_ = 0.68, *H*_*O*_ = 0.72). While our estimates are not directly comparable with those obtained from other studies employing different microsatellite loci (Table 5), they do provide the first evidence that the brown bears in the GKM in northeastern Anatolia are not genetically impoverished. Measures of genetic diversity for threatened populations, for example for the brown bear population in Spain (Table 5), are far lower than those we report here. As the main source population for brown bears in northeastern Anatolia and surrounding regions, the GKM population represents an important genetic reservoir that needs to be preserved.

**Table 5:** Comparison of genetic diversity measures between GKM and other brown bear populations.

*Population*	*N*_*allele*_^a^	*H*_*O*_	*H*_*E*_	*F*_*IS*_	*N*_*samp*_	*N*_*loc*_	Source
***Anatolian GKM***	***8.6***	***0.74***	***0.78***	***0.072***	***42***	***5***	***this study***
other populations:							
Russia (Kirov)	**8.1**	**0.83**	**0.83**	–	13	17	Tammeleht et al. (2010)
Russia (Arkhangelsk)	**7.7**	**0.78**	**0.79**	–	16	17	Tammeleht et al. (2010)
Finland N	**10.8**	**0.83**	**0.83**	−0.001	164	12	Kopatz et al. (2014)
Finland S	**8.8**	**0.79**	**0.78**	−0.012	122	12	Kopatz et al. (2014)
Romania	**7.8**	**0.72**	**0.81**	–	16	9	Zachos et al. (2008)
Romania	**8.5**	**0.76**	**0.80**	–	109	13	Straka et al. (2012)
Slovakia N	6.0	0.69	0.71	–	71	13	Straka et al. (2012)
Slovakia C	6.0	0.69	0.70	–	96	13	Straka et al. (2012)
Slovakia E	5.2	0.66	0.65	–	16	13	Straka et al. (2012)
Scandinavia M	5.8	0.65	0.66	–	88	19	Waits et al. (2000)
Scandinavia NN	5.5	0.66	0.66	–	29	19	Waits et al. (2000)
Scandinavia NS	6.2	0.66	0.66	–	108	19	Waits et al. (2000)
Scandinavia S	5.4	0.76	0.66	–	155	19	Waits et al. (2000)
Croatia	**7.6**	**0.74**	**0.75**	–	156	12	Kocijan et al. (2011)
Slovenia	6.8	0.73	0.74	–	513	20	Skrbinšek et al. (2012)
Macedonia	5.8	0.75	0.72	0.003	14	18	Karamanlidis et al. (2014a)
Serbia	5.4	0.78	0.69	–	10	16	Karamanlidis et al. (2014b)
Greece	5.6	0.65	0.69	0.059	49	10	Karamanlidis et al. (2012)
Bulgaria	8.8	0.66	0.73	–	125	13	Nowak et al. (2014)
Estonia	7.4	0.66	0.68	–	62	17	Tammeleht et al. (2010)
Italy	2.4	0.44	0.46	–	17	9	Zachos et al. (2008)
Spain W	3.3	0.44	0.45	–	39	18	Pérez et al. (2009)
Spain E	1.7	0.28	0.25	–	71	18	Pérez et al. (2009)

**Notes.**

Number of alleles (*N*_*allele*_), observed (*H*_*O*_) and expected (*H*_*E*_) heterozygosity, inbreeding coefficient (*F*_*IS*_), number of samples (*N*_*samp*_), number of loci (*N*_*loc*_).

For populations with similar genetic variation as GKM, values are highlighted in bold.

a Mean number of alleles per locus.

Unfortunately, we were not able to satisfactorily resolve population structure in our study area because we were only able to obtain a moderate number of genotypes from the Bayburt province. None of the analyses could reject the hypothesis of a single population in our study area (e.g., Fig. 2), and we did detect one bear in both provinces, indicating that (current) landscape features do not hinder the movement of brown bears between these two areas. However, due to the uneven sampling between areas, which can be problematic for some analyses of population structure (e.g., Puechmaille, 2016), we prefer to refrain from definitively stating that there is no population subdivision. Consequently, we cannot claim that the bears in the GKM can be managed as a single conservation unit. Further work is clearly needed to address this, particularly with respect to additional sampling; ideally, also increasing the geographic scope of the current study.

Genetic monitoring of this population is also important to gauge the impact of habitat loss and habitat fragmentation in the region (Şekercioğlu et al., 2011; Balkenhol & Waits, 2009). These result from the ongoing development of infrastructure (big dams, HEPPs, roads and high-voltage transmission pylons) in areas where still continuous pristine habitat exists (e.g., natural old forests) for large carnivores. Extensive loss of habitat, and concomitant fragmentation of the remaining habitat, has already been reported for Eurasian lynx, golden jackals and wild goats in the same region (Özdemirel, Turak & Bilgin, 2016). Due to the importance of the brown bear population in the GKM for on-going and future conservation actions, the impacts of habitat alterations need to be understood, and when possible minimized, in order to preserve both the current genetic variation in these brown bears and the connectivity of their populations.

### Sources of noninvasively collected samples

It was recently shown that hair samples obtained from rubbing trees perform better for genotyping than hair samples collected by other noninvasive methods, such as hair from corral traps (Berezowska-Cnota et al., 2017). We similarly observed that hair samples from rubbing trees performed best among the hair sample sources used in our study (Table 4). Our hair sampling strategy relied mostly on rubbing objects (such as trees and power poles) and barbed wire on rubbing trees. The significant reduction in genotyping success of samples collected from power poles that we observed may be due to a higher proportion of shed guard hair without follicles that got stuck to the creosote. Moreover, hair on power poles were generally exposed to direct sunlight for long periods of time, which most probably caused higher levels of DNA degradation (Stetz, Seitz & Sawaya, 2014), when compared to hair obtained from trees where direct sunlight is almost absent. While power poles with creosote should not be discounted as a source for hair samples in future studies, it is advised to supplement these and rubbing trees with other sources from which samples could be collected noninvasively. For example, adding some barbed wire to power poles may increase the retention of underfur hair.

As reported for North American bears (Lamb, Walsh & Mowat, 2016), rubbing trees in the GKM also appear to be mostly used by male bears as only 12 of 71 genotypes belongs to female bears (Table S1). Thus, population estimates relying on noninvasive sampling from rubbing trees will be male-biased. As we detected discrepancies in the sexes of individuals when using only SRY, using a more sophisticated sexing procedure (e.g., a multilocus approach with internal controls/validation, Sastre et al., 2009) would be safer for noninvasive samples to determine sex of individuals. Considering this, and the fact that female brown bears in Turkey have very small home ranges (Ambarlı, 2012), greater effort needs to be allocated to sample female bears with cubs, as well as sub-adult bears. This may be accomplished by including fecal samples in the analyses or by establishing hair traps in more remote areas.

## Conclusions

Any conservation action plan for brown bears in the Lesser Caucasus needs to consider the influences of big dam and HEPP constructions on this genetically highly diverse bear population (high mtDNA diversity (Çilingir et al., 2016) and nuclear diversity (this study)). Therefore, we would like to advocate the construction of conservation corridors over reservoirs of big dams or major roads to decrease the severity of genetic isolation and habitat fragmentation. In this way, brown bears in Turkey might continue to serve as a genetic reserve for southwest Asia (e.g., not only for Lesser Caucasus, but also for the Middle East). We suggest long term genetic monitoring of this valuable bear population by using at least two noninvasive sampling techniques to include female bears. We also urge authorities to plan a series of conservation measures in order to guarantee the gene flow needed between subpopulations in order to maintain sustainable levels of genetic diversity.

##  Supplemental Information

10.7717/peerj.5660/supp-1 Figure S1 Structure plots for *K* = 1 to *K* = 6

10.7717/peerj.5660/supp-2 Figure S2 Plot of DIC against the number of *K* clusters, and Tess plots for *K* = 2 to *K* = 6

10.7717/peerj.5660/supp-3 Table S1 Primers (name, sequence and source) used in this study

10.7717/peerj.5660/supp-4 Supplemental Information 1 Genotypes used for the analysis

## Additional Information and Declarations

### Data Availability

The following information was supplied regarding data availability: The genotypes used for further analysis are provided as a Supplemental File.

## References

[ref-1] Adamack AT, Gruber B (2014). PopGenReport: simplifying basic population genetic analyses in R. Methods in Ecology and Evolution.

[Ambarlı(2012] Ambarlı H (2012). Spatio-temporal ecology, habitat use and population size of brown bears (*Ursus arctos*) in Yusufeli, Turkey. PhD thesis.

[Ambarlı(2016] Ambarlı H (2016). Litter size and basic diet of brown bears (*Ursus arctos*, Carnivora) in northeastern Turkey. Mammalia.

[ref-4] Ambarlı H, Ertürk A, Soyumert A (2016). Current status, distribution, and conservation of brown bear (Ursidae) and wild canids (gray wolf, golden jackal, and red fox; canidae) in Turkey. Turkish Journal of Zoology.

[ref-5] Atak E, Öztok D (2013). 10 soruda Hidroelektrik Santraller.

[ref-6] Balkenhol N, Waits LP (2009). Molecular road ecology: exploring the potential of genetics for investigating transportation impacts on wildlife. Molecular Ecology.

[ref-7] Bellemain E, Nawaz MA, Valentini A, Swenson JE, Taberlet P (2007). Genetic tracking of the brown bear in northern Pakistan and implications for conservation. Biological Conservation.

[ref-8] Berezowska-Cnota T, Luque-Márquez I, Elguero-Claramunt I, Bojarska K, Okarma H, Selva N (2017). Effectiveness of different types of hair traps for brown bear research and monitoring. PLOS ONE.

[ref-9] Bowcock AM, Ruiz-Linares A, Tomfohrde J, Minch E, Kidd JR, Cavalli-Sforza LL (1994). High resolution of human evolutionary trees with polymorphic microsatellites. Nature.

[ref-10] Bull JK, Heurich M, Saveljev AP, Schmidt K, Fickel J, Förster DW (2016). The effect of reintroductions on the genetic variability in Eurasian lynx populations: the cases of Bohemian-Bavarian and Vosges-Palatinian populations. Conservation Genetics.

[ref-11] Chakraborty R, De Andrade M, Daiger SP, Budowle B (1992). Apparent heterozygote deficiencies observed in DNA typing data and their implications in forensic applications. Annals of Human Genetics.

[ref-12] Çilingir FG, Akin Pekşen Ç, Ambarli H, Beerli P, Bilgin CC (2016). Exceptional maternal lineage diversity in brown bears (*Ursus arctos*) from Turkey. Zoological Journal of the Linnean Society.

[ref-13] Chen C, Durand E, Forbes F, François O (2007). Bayesian clustering algorithms ascertaining spatial population structure: a new computer program and a comparison study. Molecular Ecology Resources.

[ref-14] Conover MR (2001). Resolving human-wildlife conflicts: the science of wildlife damage management.

[ref-15] Şekercioğlu ÇH, Anderson S, Akçay E, Bilgin R, Can ÖE, Semiz G, Tavşanoğlu Ç, Yokeş MB, Soyumert A, İpekdal K, Sağlam İK, Yücel M, Nüzhet Dalfes H (2011). Turkey’s globally important biodiversity in crisis. Biological Conservation.

[ref-16] Excoffier L, Lischer HE (2010). Arlequin suite ver 3.5: a new series of programs to perform population genetics analyses under Linux and Windows. Molecular Ecology Resources.

[ref-17] Falush D, Stephens M, Pritchard JK (2003). Inference of population structure using multilocus genotype data: linked loci and correlated allele frequencies. Genetics.

[ref-18] Frankham R (2005). Genetics and extinction. Biological Conservation.

[ref-19] Frosch C, Dutsov A, Zlatanova D, Valchev K, Reiners TE, Steyer K, Pfenninger M, Nowak C (2014). Noninvasive genetic assessment of brown bear population structure in Bulgarian mountain regions. Mammalian Biology.

[ref-20] Galpern P, Manseau M, Hettinga P, Smith K, Wilson P (2012). Allelematch: an R package for identifying unique multilocus genotypes where genotyping error and missing data may be present. Molecular Ecology Resources.

[ref-21] Galpern P, Peres-Neto PR, Polfus J, Manseau M (2014). MEMGENE: spatial pattern detection in genetic distance data. Methods in Ecology and Evolution.

[ref-22] Goudet J (2002). http://www2.unil.ch/popgen/softwares/fstat.htm.

[ref-23] IUCN (2016). Ursus arctos. http://dx.doi.org/10.2305/IUCN.UK.2017-3.RLTS.T41688A121229971.en.

[ref-24] Jakobsson M, Rosenberg NA (2007). CLUMPP: a cluster matching and permutation program for dealing with label switching and multimodality in analysis of population structure. Bioinformatics.

[ref-25] Jombart T (2008). Adegenet: a R package for the multivariate analysis of genetic markers. Bioinformatics.

[ref-26] Karamanlidis AA, Paunović M, Ćirović D, Karapandža B, Skrbinšek T, Zedrosser A (2014b). Population genetic parameters of brown bears in western Serbia: implications for research and conservation. Ursus.

[ref-27] Karamanlidis AA, Stojanov A, Hernando MG, Ivanov G, Kocijan I, Melovski D, Skrbinšek T, Zedrosser A (2014a). Distribution and genetic status of brown bears in FYR Macedonia: implications for conservation. Acta Theriologica.

[ref-28] Karamanlidis AA, Straka M, Drosopoulou E, Hernando MG, Kocijan I, Paule L, Scouras Z (2012). Genetic diversity, structure, and size of an endangered brown bear population threatened by highway construction in the Pindos Mountains, Greece. European Journal of Wildlife Research.

[ref-29] Kocijan I, Galov A, Ćetković H, Kusak J, Gomerčić T, Huber Ð (2011). Genetic diversity of Dinaric brown bears (*Ursus arctos*) in Croatia with implications for bear conservation in Europe. Mammalian Biology.

[ref-30] Kopatz A, Eiken HG, Aspi J, Kojola I, Tobiassen C, Tirronen KF, Danilov PI, Hagen SB (2014). Admixture and gene flow from Russia in the recovering Northern European brown bear (*Ursus arctos)*. PLOS ONE.

[ref-31] Kosman E, Leonard KJ (2005). Similarity coefficients for molecular markers in studies of genetic relationships between individuals for haploid, diploid, and polyploid species. Molecular Ecology.

[ref-32] Lamb, Walsh D, Mowat G (2016). Factors influencing detection of grizzly bears at genetic sampling sites. Ursus.

[ref-33] Lortkipanidze B (2010). Brown bear distribution and status in the South Caucasus. Ursus.

[ref-34] Miller CR, Joyce P, Waits LP (2002). Assessing allelic dropout and genotype reliability using maximum likelihood. Genetics.

[ref-35] Murtskhvaladze M, Gavashelishvili A, Tarkhnishvili D (2010). Geographic and genetic boundaries of brown bear (*Ursus arctos*) population in the Caucasus. Molecular Ecology.

[ref-36] Nowak C, Domokos C, Dutsov A, Frosch C (2014). Molecular evidence for historic long-distance translocations of brown bears in the Balkan region. Conservation Genetics.

[ref-37] Özdemirel KB, Turak AS, Bilgin CC (2016). Impact of large scale dam construction on movement corridors of mammals in Artvin, north-eastern Turkey. Applied Ecology and Environmental Research.

[ref-38] Paetkau D, Waits LP, Clarkson PL, Craighead L, Vyse E, Ward R, Strobeck C (1998). Variation in genetic diversity across the range of north American brown bears. Conservation Biology.

[ref-39] Paradis E, Claude J, Strimmer K (2004). APE: analyses of phylogenetics and evolution in R language. Bioinformatics.

[ref-40] Pérez T, Vázquez F, Naves J, Fernández A, Corao A, Albornoz J, Domínguez A (2009). Non-invasive genetic study of the endangered Cantabrian brown bear. Conservation Genetics.

[ref-41] Pritchard JK, Stephens M, Donnelly P (2000). Inference of population structure using multilocus genotype data. Genetics.

[ref-42] Puechmaille SJ (2016). The program structure does not reliably recover the correct population structure when sampling is uneven: subsampling and new estimators alleviate the problem. Molecular Ecology Resources.

[ref-43] R Core Team (2015). https://www.R-project.org/.

[ref-44] Roon D, Waits L, Kendall K (2003). A quantitative evaluation of two methods for preserving hair samples. Molecular Ecology Notes.

[ref-45] Rosenberg NA (2004). DISTRUCT: a program for the graphical display of population structure. Molecular Ecology Resources.

[ref-46] Sastre N, Francino O, Lampreave G, Bologov VV, López-Martín JM, Sánchez A, Ramírez O (2009). Sex identification of wolf (*Canis lupus*) using non-invasive samples. Conservation Genetics.

[ref-47] Skrbinšek T, Jelenčić M, Waits LP, Potočnik H, Kos I, Trontelj P (2012). Using a reference population yardstick to calibrate and compare genetic diversity reported in different studies: an example from the brown bear. Heredity.

[ref-48] Stetz JB, Seitz T, Sawaya MA (2014). Effects of exposure on genotyping success rates of hair samples from brown and American black bears. Journal of Fish and Wildlife Management.

[ref-49] Straka M, Paule L, Ionescu O, Štofík J, Adamec M (2012). Microsatellite diversity and structure of Carpathian brown bears (*Ursus arctos*): consequences of human caused fragmentation. Conservation Genetics.

[ref-50] Swenson JE, Taberlet P, Bellemain E (2011). Genetics and conservation of European brown bears *Ursus arctos*. Mammal Review.

[ref-51] Tammeleht E, Remm J, Korsten M, Davidson J, Tumanov I, Saveljev A, Mannil P, Kojola I, Saarma U (2010). Genetic structure in large, continuous mammal populations: the example of brown bears in northwestern Europe. Molecular Ecology.

[ref-52] Tsaparis D, Karaiskou N, Mertzanis Y, Triantafyllidis A (2015). Non-invasive genetic study and population monitoring of the brown bear (*Ursus arctos*) (Mammalia: Ursidae) in Kastoria region—Greece. Journal of Natural History.

[ref-53] Valière N (2002). GIMLET: a computer program for analysing genetic individual identification data. Molecular Ecology Resources.

[ref-54] Van Oosterhout C, Hutchinson WF, Wills DPM, Shipley P (2004). MICRO-CHECKER: software for identifying and correcting genotyping errors in microsatellite data. Molecular Ecology Notes.

[ref-55] Waits LP, Taberlet P, Swenson JE, Sandergren F, Franzen R (2000). Nuclear analysis of genetic diversity and gene flow in the Scandinavian brown bear (*Ursus arctos*). Molecular Ecology.

[ref-56] WWF-Türkiye (2015). Yeşil Yol Karadeniz’i yoldan çıkaracak.

[ref-57] Yeh FC, Yang RC, Boyle TBJ, Ye ZH, Mao JX (1997). https://sites.ualberta.ca/ fyeh/popgene.html.

[ref-58] Zachos FE, Otto M, Unici R, Lorenzini R, Hartl GB (2008). Evidence of a phylogeographic break in the Romanian brown bear (*Ursus arctos*) population from the Carpathians. Mammalian Biology.

