# Supplementary figures and images for: Population genetics of the main population of brown bears in southwest Asia
Source: PeerJ. 2018 Sep 21;6:e5660. doi: 10.7717/peerj.5660 (PMC6152452; doi:10.7717/peerj.5660)

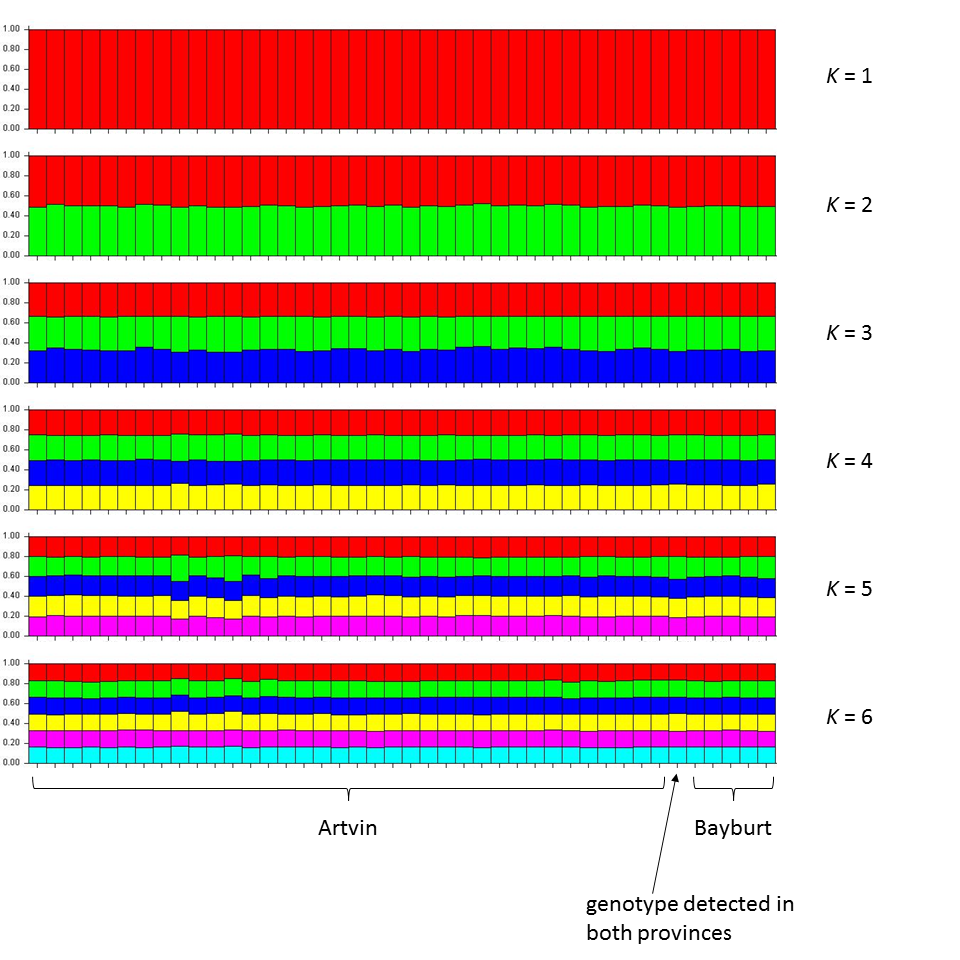

Supplement: Figure S1 [file peerj-06-5660-s001.png]

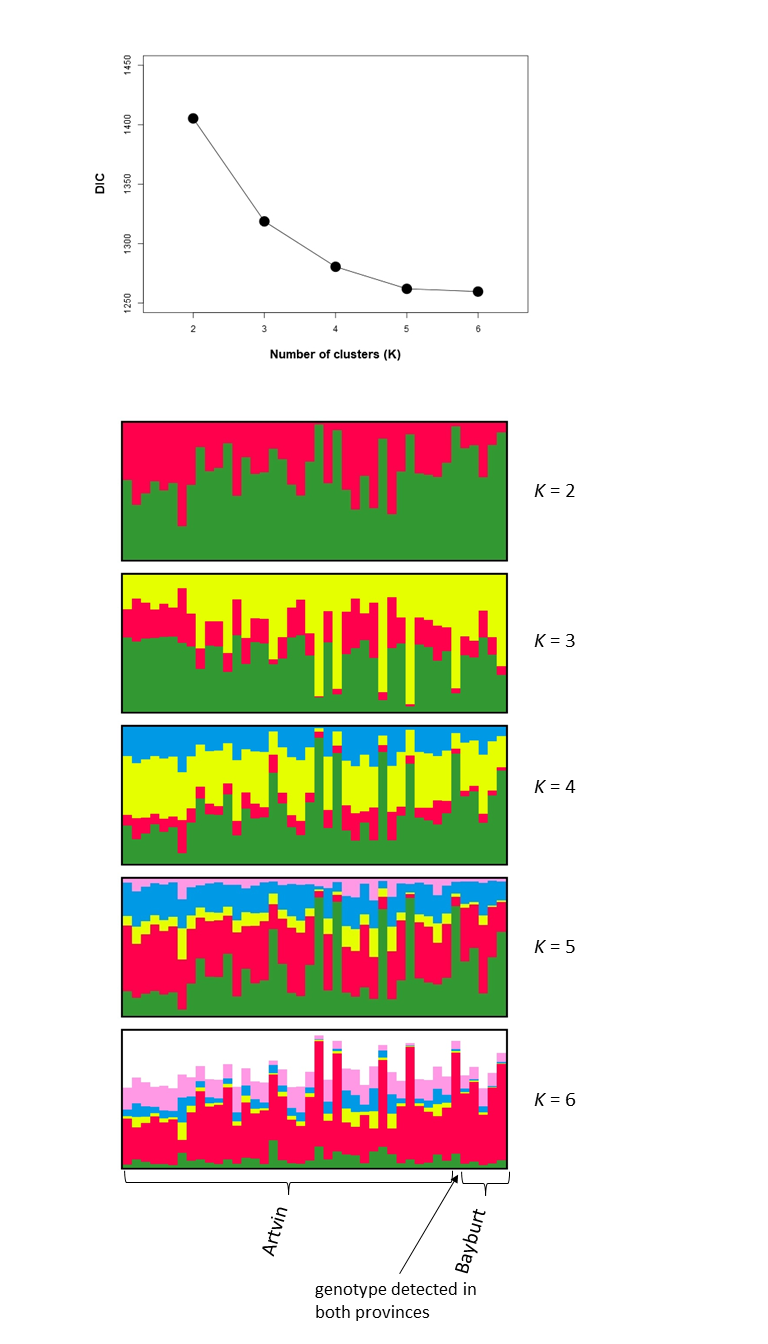

Supplement: Figure S2 [file peerj-06-5660-s002.png]
