# Supplementary material for: Population genetics of the main population of brown bears in southwest Asia
Source: PeerJ. 2018 Sep 21;6:e5660. doi: 10.7717/peerj.5660 (PMC6152452; doi:10.7717/peerj.5660)
Supplement: Table S1 [file peerj-06-5660-s003.docx]

**Table S1.** Primers (name, sequence and source) used in this study

| **Primer** | **Sequence** | **Dye** | **Mix** | **Source** |
| --- | --- | --- | --- | --- |
| G10C | F: AAGCAGAAGGCCTTGATTTCCTG  R: GGGACATAAACACCGAGACAGC | 6-FAM | 1 | Paetkau et al., 1995 |
| UarD1585 | F: CATGTGTCGTGATTTCCGTC  R: ACCCAAATGTCTATGGGCAG | HEX | 1 | Kleven et al., 2012 |
| G1D | F: TCTCTTTTCCTTTAGGGGACTC  R: CTAGCACCCAGCAAGGTATAATA | NED | 1 | Paetkau & Strobeck 1994 |
| SRY | F: GAACGCATTCTTGGTGTGGTC  R: TGATCTCTGAGTTTTGCATTTG | 6-FAM | 2 | Taberlet et al., 1997 |
| Mu50 | F: GTCTCTGTCATTTCCCCATC  R: ACCTGGAACAAAAATTAACAC | NED | 2 | Bellemain & Taberlet, 2004 |
| UarT739 | F: AATTGCCAAAGGTCATGGTC  R: AGCCAAAGTATGGAAGCAGC | 6-FAM | 2 | Kleven et al., 2012 |
| Mu05 | F: ATGTGGATACAGTGGAATAGACC  R: GTTTCTTGTGACATGAACTGAAACTTGTTAT | HEX | 2 | Taberlet et al., 1995 |
| UarD3139 | F: CTGGGACTCAAATCAATGTCTG  R: ATCCGTTCATCAACCAAAGG | 6-FAM | 3 | Kleven et al., 2012 |
| UarD3684 | F: AAAAGCCCAGATGTCTGACG  R: ATTGCAAATGGCAGGATTTC | NED | 3 | Kleven et al., 2012 |
| G10X | F: CCCTGGTAACCACAAATCTCT  R: TCAGTTATCTGTGAAATCAAAA | HEX | 3 | Paetkau et al., 1995 |
| Mu23 | F: GCCTGTGTGCTATTTTATCC  R: TAGACCACCAAGGCATCAG | 6-FAM | 3 | Bellemain & Taberlet, 2004 |
|  |  |  |  |  |
